# Partners and Ex-Partners in Dreams: A Diary Study

**DOI:** 10.3390/clockssleep3020018

**Published:** 2021-05-26

**Authors:** Michael Schredl, Lara C. Wood

**Affiliations:** Central Institute of Mental Health, Medical Faculty Mannheim, Heidelberg University, Postfach 12 21 20, 68072 Mannheim, Germany; lara.wood@hotmail.de

**Keywords:** dreaming, romantic relationships, partner, ex-partner

## Abstract

Romantic relationships are an important part of human life and thus, according to the continuity hypothesis of dreaming, one’s romantic partner should show up in dreams quite frequently. The present study is based on 1612 dream reports provided by 425 students. The findings confirmed the hypothesis that partner dreams are more frequent than ex-partner dreams and, thus, support the continuity hypothesis of dreaming. Moreover, interactions with ex-partners within the dream were more often negatively toned compared to dreamed interactions with the partner. Unexpectedly, we also found more positive emotions and friendliness in ex-partner dreams compared to partner dreams, indicating that partner dreams are more mundane. To conclude, dreams reflect important aspects of romantic partnerships and their break-ups and, thus, can be very helpful in psychotherapy.

## 1. Introduction

Romantic relationships are an important part of human life [1]. Based on the continuity hypothesis of dreaming [2,3], one’s romantic partner should show up quite frequently in dreams since emotionally intense experiences are more likely to be incorporated into dreams [4,5]. Indeed, single-case studies [6,7], dream content analytic studies [8,9,10], and studies using retrospective estimates about the frequency of partner dreams in relation to all remembered dreams [11,12] showed that about 20 to 30% of all dreams of persons with stable partnerships include the partner. Moreover, the amount of time spent with the partner in waking life was correlated positively with the percentage of partner dreams [12,13].

Analyzing a long dream series recorded over a time period of 30 yrs (including nine different partnerships) indicated that relationship duration and emotional intensity was related to the percentage of partner dreams—with less frequent partner dreams during short and less intense partnerships [14]. Interestingly, systematic studies investigating the content of partner dreams are very scarce; solely Schredl [15] reported, for a single case, the types of activities the dreamer and the partner were engaged in within the dream: shared activities, e.g., driving in a car, visiting someone, were the most common topic (ca. 43%); meeting/talking to each other (ca. 26%) and erotic activities (ca. 20%) were less frequent. The topic of separation or being separated was very rare (ca. 3%).

Whereas the emotional tone of the partner dreams was balanced in the single case study [15], the retrospectively estimated emotional tone of partner dreams was positive, especially compared to the estimated emotional tone of all remembered dreams [12]. The cross-sectional finding that subjective partnership quality is related to the emotional tone of partner dreams [12] was confirmed by a longitudinal study using a diary protocol over a two-week period, i.e., after days with high intimacy, the partner dreams were more positive and less negative [16]. Moreover, there was also an effect of partner dreams that included jealousy or conflict on the subsequent day; negative emotions in the dream were followed by days with more conflict within the relationship [16]. A more extreme effect can be found in intimate partner violence survivors who experience nightmares related to the abusive relationship [17].

On the other hand, sharing dreams is related to partnership intimacy [18]; this cross-sectional finding was confirmed by an experimental study that sharing dreams regularly increased the intimacy between partners [19]. Similarly, working with dreams in couples (one dream of each partner) benefited the female partner especially [20]. To summarize, despite the relatively sparse research in the area, partner dreams reflect the waking-life partnership on the one hand and, on the other, can also have an effect on the partnership, i.e., waking and dreaming are intertwined.

Only a few studies [6,12,14,15,21,22] investigated dreams including an ex-partner systematically. The decrease in the frequency of dreams with the partner (the ex-partner) in longitudinal single-case studies [14,15] seems plausible; however, even years after the separation, about 5% of the dreams included the ex-partner [15,21]. In a cross-sectional study [12], the estimated percentage of ex-partner dreams (ca. 8%) was much lower compared to the estimated percentage of partner dreams (ca. 21%)—confirming the single-case studies’ findings. The emotional tone of the ex-partner dreams was more negative when compared with the partner dreams but still balanced regarding negative and positive emotions [12]; nevertheless, single-case studies [21,22] showed more negative emotions and aggression in the dreamed interactions with the ex-partner, even after 20 yrs [21].

Regarding the content of ex-partner dreams, erotic topics and meeting/talking to the partner/ex-partner were equally frequent in comparison to partner dreams but shared activities were less common in ex-partner dreams, whereas being separated was more common in ex-partner dreams compared to partner dreams [15]. Overall, the findings that ex-partner dreams are less frequent than partner dreams seem plausible as the time spent together in waking-life is typically reduced drastically. The ex-partner dreams might also reflect the conflict and pain that is often associated with break-ups of romantic relationships [1]. However, the database in this area is quite small.

The present analysis of dream reports collected within the context of a study on dream recall [23] investigated the frequency and contents of dreams including the partner and/or ex-partner of the dreamer. Based on the continuity hypothesis, we expected that partner dreams are more frequent than ex-partner dreams. Moreover, we expected more negatively toned interactions with the ex-partner within the dream compared to dreamed interactions with the partner.

## 2. Method

### 2.1. Participants

Overall, 425 persons (361 women and 64 men) with a mean age of 23.40 ± 5.41 years (range: 16 to 61 years; two missing values) participated in the present study, mostly psychology students. The participants reported 3.79 ± 1.36 dreams with a range from 1 to 10 dreams. The mean length of the 1612 dream reports was 148.98 words ± 98.90 with a range from 11 to 654.20.

### 2.2. Dream Diary

The participants kept a standardized dream diary for 14 consecutive days. If able to recall at least one dream, participants were asked to record their dream(s) as completely as possible. In order to avoid decrease in motivation in high recallers, participants were asked to record their dreams only on the first five mornings with successful dream recall. After that, they only checked whether they recalled a dream or not.

### 2.3. Dream Content Analysis

First, the dream reports (all dreams reported on one morning were combined) were scored regarding the occurrence of partner (Yes/No) and/or ex-partner (Yes/No). In addition, it was coded as to whether the partner or ex-partner within the dream was identifiable as fictitious, i.e., not the waking-life partner or ex-partner of the dreamer (examples see Appendix A). The interrater reliability (Cohen’s kappa) for the partner scale was κ = 0.902; for the ex-partner, κ = 0.918 (see procedure section).

In a second step, all interactions between dreamer and partner and/or ex-partner within the dream were coded using two different rating systems. If within the dream or different dream reports of the same night, several interactions with the partner or ex-partner occurred, these interactions were coded separately. Each interaction was coded for the presence of the following categories: erotic activities (kissing, physical intimacy, sex), shared activities (travelling, going to the movies, attending a party, shopping), meeting each other including talking, seeing each other without further contact, thinking about the partner/ex-partner (also includes talking about the partner/ex-partner with others). In addition, it was coded whether or not the relationship status (being separated) was explicitly mentioned within the dream. The emotions between the dreamer and partner/ex-partner were rated on a four-point scale (−1 = negative, 0 = neutral/no explicit mentioned emotions, +1 = positive emotions, +2 = positive and negative emotions). Examples of situations with negative emotions are: jealousy, feeling hurt by seeing the partner/ex-partner with a new partner, quarreling, and partner/ex-partner rejecting the dreamer. The following positive emotions were reported: enjoying physical intimacy, the joy of seeing partner/ex-partner, intense conversation, and partner/ex-partner is helping the dreamer. For statistical analyses, the emotion scale was re-coded into two binary variables: occurrence of positive emotions (Yes/No) and occurrence of negative emotions (Yes/No). The interrater reliability coefficients for the scales were as follows: κ = 0.877 (erotic activities), κ = 0.705 (sharing activities), κ = 0.731 (meeting/talking), κ = 0.655 (seeing the partner/ex-partner), κ = 0.550 (thinking about the partner/ex-partner), κ = 0.791 (emotions), and κ = 0.658 (relationship status)

In addition, social interactions between the dreamer and partner/ex-partner within the dream were coded according to the rules given by Hall and Van de Castle [24]. While the precise coding rules for each topic are found in Hall and Van de Castle [24] and Domhoff [25], the following is a short summary. Aggressive interactions are scored for the dreamer being the aggressor and/or being the victim, and for intensity on an 8-point nominal scale (ranging from 1 = aggressive thoughts to 8 = aggressive acts resulting in death). Aggressions coded from 1 (aggressive thoughts) to 4 (accusation/verbal threat) were grouped into non-physical aggression, and aggressions coded from 5 (theft/destruction) to 8 (aggressive acts resulting in death) into physical aggression. Friendly interactions are scored on a 7-point subscale (ranging from 1 = friendly feelings to 7 = the desire for a long-term relationship) and were also coded for the dreamer initiating the friendly interaction and the dreamer being the recipient of friendly interactions. Lastly, sexuality was coded on a 5-point scale (ranging from 1 = sexual thoughts to 5 = sexual intercourse). The interrater reliability coefficients for the scales were as follows: κ = 0.698 (non-physical aggression), κ = 0.662 (physical aggression), κ = 0.636 (dreamer is aggressive), κ = 0.531 (partner/ex-partner is aggressive), κ = 0.596 (friendly interaction), κ = 0.791 (dreamer is friendly), κ = 0.398 (partner/ex-partner is friendly), and κ = 0.935 (sexuality).

### 2.4. Procedure

The dream reports were collected within a study entitled “Sleep, dreams, and personality” that was designed to investigate factors related to home dream recall [23]. The students were approached after their lectures and invited to participate. They received course credits or alternatively, a small monetary compensation. For these types of studies (questionnaire studies with psychology students) ethics approval was at the time the study was carried out (in 2000) not obligatory. Participants were informed regarding the study aims and gave consent by returning their materials. Due to the intense recruiting strategy, the majority of the first-year psychology students of the three universities of Heidelberg, Landau, and Mannheim participated. Exclusion criteria were not formulated for the study. The participants completed several questionnaires (personality, sleep quality, stress, and creativity) and maintained a dream diary. Of the 444 participants, 425 reported at least one dream during the study period of two weeks. The dream reports were typed, randomized regarding their order, and coded by an external judge for the presence of partner and/or ex-partner. A second judge checked the codings and—in case of a mismatch—resolved these differences with a third judge and also, using the gender of the dreamer as additional information, assuming most relationships were heterosexual (not available to the first judge).

The second judge coded all the partner/ex-partner interactions within the dreams along the scales described in the dream content analysis section. Another judge independently coded 92 interactions (89 dreams) in order to compute interrater reliability coefficients (Cohen’s kappa) [26]. The strength of agreement is fair for kappa values from 0.21 to 0.40, moderate for 0.41 to 0.60, substantial for 0.61 to 0.80, and almost perfect for values greater than 0.81 [27]. A slightly different interpretation of kappa values was published by McHugh [28]: minimal (0.21–0.39), weak (0.40–0.59), moderate (0.60–0.79), strong (0.80–0.90), and almost perfect (above 0.90).

Statistical analysis was performed using SAS for Windows 9.4 (SAS Institute Cary, Cary, NC, USA). In order to test a possible difference in the frequencies of partner vs. ex-partner dreams, we computed the number of each dream type for each participant and compared these figures using a Wilcoxon test for paired samples, as these variables were not normally distributed. The variables related to the interaction between dreamer and partner/ex-partner within the dreams were analyzed using the GLIMMIX procedure to account for the fact that participants could contribute multiple observations. For one variable (seeing the partner/ex-partner), the variance in the RANDOM statement (accounting for the interdependence within subject) was zero, and thus, the analysis for this variable was carried out without the RANDOM term.

## 3. Results

Overall, 273 dreams included the partner of the dreamer (see Table 1). In an additional nine cases, the dreamer had a partner within the dream but the dream content included information that indicated this was not the waking-life partner of the dreamer. An ex-partner showed up in 77 dreams. Additionally, one dream included a fictitious ex-partner. Within this dream sample, 17 dreams included the partner and the ex-partner of the dreamers.

Of the 425 participants, 39.06% reported at least one partner dream and 15.76% reported at least one ex-partner dream. The difference between the number of partner dreams per participant and the number of ex-partner dreams per participant was significant (Wilcoxon test: z = 8.5, *p* < 0.0001, effect size = 1.293), i.e., partner dreams were much more common than ex-partner dreams (see Table 2).

As expected, the interactions with the ex-partner within the dream were more often negatively toned compared to the interactions with the partner (see Table 3). However, there were also more positive and more erotic interactions with ex-partners than with the partner. Sharing an activity with the partner was more common than sharing an activity with the ex-partner (see Table 3), whereas dreams in which the ex-partner was seen but not directly contacted were more frequent than dreams in which the partner was seen. Thinking about the partner occurred as often as thinking about the ex-partner. Explicit mentioning of being separated was more common in ex-partner dreams compared to partner dreams (see Table 3). The ratings based on the Hall and Van de Castle rating system indicated that aggression, friendliness, and sexuality were more often found in the interaction with the ex-partner compared to the interactions with the partner (see Table 3). Only the coding regarding the dreamer being aggressive did not show a significant difference.

## 4. Discussion

The findings confirmed the hypothesis that partner dreams are more frequent than ex-partner dreams and, thus, support the continuity hypothesis of dreaming. Moreover, interactions with ex-partners within the dream were more often negatively toned compared to dreamed interactions with the partner—a finding that is also in line with the continuity hypothesis of dreaming. Unexpectedly, we also found more positive emotions and friendliness in ex-partner dreams compared to partner dreams, indicating that partner dreams might often include more mundane topics such as sharing activities with the partner with no explicitly mentioned interactions between the partners. Due to methodological issues (no information about relationship status, relationship duration, etc.), these first findings can inform future research projects.

Prior to discussing the findings in detail, a few methodological issues must be addressed. As the dreams were collected in a study addressing another research question (factors of home dream recall), relationship status and variables such as relationship duration or the time interval after separation in singles were not elicited. Despite this drawback, the findings (percentage of partner dreams and ex-partner dreams) do match those of other studies [10,12] that included that information. Particularly in the context of the ex-partner dreams, it would be interesting to know more about the separation such as who broke up the relationship or how are the emotions regarding the ex-partner in waking-life—as the findings suggest that at least some dreamers might also experience positive emotions towards their ex-partner. As the relationship status of the participants was not known, we did not analyze, for example, gender differences in men and women regarding their percentage of partner and ex-partner dreams (as the percentage of participants being in partnership might differ). In addition, the number of interactions with the partner (N = 22) and ex-partner (N = 9) in men’s dreams was relatively small, thus statistical comparisons regarding gender differences would be largely underpowered.

Another methodological issue is the reliability of the dream content analytic scales. Although identifying the partner in a dream is not easy (in German “Mein Freund/Meine Freundin”) as this expression can also be used to describe a particular friend, the kappa of this scale (partner present vs. not present) was very high. Although the interrater reliability coefficients were lower for the interaction scales, the values were still within an acceptable range [27,28]. One might expect that more extensive training of the external judges might be beneficial, at least for some scales [29], but systematic studies in these areas are scarce.

The percentage of partner dreams in this sample of 425 psychology students was high (about 17%). In a previous study [10], the percentage of students in a stable partnership was about 57%—a finding that was confirmed by large-scale surveys [30]. If the proportion of persons with stable partnerships and singles was comparable in the present sample, the estimated partner dream percentage in persons with partnership would increase to 29.72%, which would be in line with previous dream diary studies carried out in student samples [8,9,10]—again, supporting the validity of the present findings. This high percentage of partner dreams supports the continuity hypothesis, as close persons who are very important to the dreamer and with whom the dreamer has spent a lot of time with in his or her waking-life are often found in dreams.

The content analysis of partner dreams confirmed the findings of a single case study that most partner dreams (about 70%) featured shared activities such as doing something together, whereas erotic interactions were relatively rare (about 8%). The negative emotions are slightly more common than positive emotions, but friendly interactions outweigh aggressive interactions. In order to follow-up on these findings, it would be necessary to elicit partnership quality such as intimacy levels or frequency of conflicts. In student samples, the aggression per character (all dream characters) varied between 0.34 (male dreamers) and 0.24 (female dreamers) [25], i.e., the interactions with the partner were less aggressive compared to the interaction with other dream characters; physical aggression was especially low. The percentage of physical aggression to all aggressive interactions was very low (about 6%) in the partner dreams compared to students’ dreams in general: 50% in male students and 34% in female students [24]. This indicates that the relationship to the romantic partner is an intimate and trusting one and not primarily conflict-laden. On the other hand, friendliness per character and sexual interactions per characters are comparable to those found in the sample of 1000 students’ dreams collected by Hall and Van de Castle [24]. Interestingly, the dreams including a fictitious romantic partner were quite scarce, especially compared to the figures of 8% or more reported by Schredl [10] and Schredl, Cadiñanos Echevarria, Saint Macary and Weiss [12]. In future studies, it would be very interesting to include a measure that elicits the attitude of the dreamer concerning not having a partner to answer the question as to whether singles who long for a (new) partnership dream more often about an as-yet fictitious partner.

In line with the continuity hypothesis of dreaming [3], the percentage of ex-partner dreams was much lower than the percentage of partner dreams—similar to the longitudinal single case study [6] and the cross-sectional study using retrospective estimates of partner and ex-partner dream frequencies [12]. It seems very plausible that the dreamer spends much less time with an ex-partner compared to the current partner. It would be very interesting to study whether factors such as total cessation of contact [6] or painfulness of the break-up have different effects on the frequency of ex-partner dreams.

As expected and shown by single-case studies [21,22], ex-partner dreams included more negatively toned interactions and more aggression. Additionally, the topic of separation is more common in ex-partner dreams. This is likely related to the conflicts that underlie the break-up or the break-up itself. However, we also found more positive emotions, friendliness, erotic activities, and sexuality in ex-partner dreams compared with partner dreams. One line of thinking is related to the fact that ex-partner dreams are less frequent than partner dreams; because the waking-life encounters with the ex-partner occurred longer ago than encounters with the current partner and that intense previous experiences still have a chance to show up even after longer time intervals [31], it might be that ex-partner dreams—if they occur—are therefore emotionally more intense (including more often negative but also positive emotions) compared to everyday partner dreams. This is in line with the findings that partner dreams include mundane, everyday activities with the partner (doing stuff together) and that these activities are less frequent in the context of ex-partners. The positive interactions with the ex-partner might be related to feelings of longing for the ex-partner, possibly wishing for a reunion. It would be interesting to study the waking-life attitude towards the ex-partner and the current relationship status of the dreamer in relation to the contents and emotions of ex-partner dreams.

To conclude, dreams reflect important aspects of romantic partnerships and their break-ups. As this analysis of a previously existing dream sample did not include any specific information about the relationship status and the relationship characteristics, further research is needed. However, even without this information, it could be shown that partner dreams are frequent and that ex-partner dreams include more negative emotions and aggression than partner dreams.

## Figures and Tables

**Table 1:** Partner and ex-partner dreams (N = 1612 dreams).

Topic	Frequency	Percentage
Partner dreams	273	16.94%
Ex-partner dreams	77	4.78%
Dreams with fictitious partner	9	0.56%
Dreams with fictitious ex-partner	1	0.06%

**Table 2:** Number of partner and ex-partner dreams per person (N = 425 participants).

Topic	Mean ± SD
Partner dreams	0.64 ± 0.95
Ex-partner dreams	0.18 ± 0.46
Number of reported dreams	3.79 ± 1.36

**Table 3:** Interactions between dreamer and partner and ex-partner within the dream.

Topic	Partner Interaction (N = 296)	Ex-Partner Interactions (N = 83)	Statistical Test	
			*t* =	*p* =
Negative emotions	24.32%	48.19%	−3.9	0.0001
Positive emotions	13.85%	26.51%	−2.7	0.0084
Erotic interaction	7.77%	26.51%	−2.2	0.0267
Sharing an activity	70.95%	56.63%	2.4	0.0165
Verbal interaction	28.04%	44.58%	−2.7	0.0078
Seeing the partner/ex-partner	5.41%	19.28%	−3.8	0.0002 *
Thinking about the partner/ex-partner	26.35%	25.30%	0.4	0.7100
Being separated (explicitly mentioned)	1.01%	26.51%	−5.5	0.0001
Total aggression	12.50%	27.71%	−3.1	0.0022
Non-physical aggression	12.16%	24.10%	−2.5	0.0128
Physical aggression	0.68%	6.02%	−2.6	0.0111
Dreamer being aggressive	8.78%	13.25%	−1.2	0.2484
Partner/Ex-partner being aggressive	8.11%	20.48%	−2.9	0.0043
Friendliness	23.31%	40.96%	−2.9	0.0038
Dreamer being friendly	18.92%	31.33%	−2.3	0.0246
Partner/Ex-partner being friendly	14.86%	25.30%	−2.1	0.0333
Sexual interaction	3.72%	12.05%	−2.4	0.0162

* Variance in the Random statement of Glimmix was zero, so the variable (participant code) was removed.

## Data Availability

The dream content analysis data are available from the first author on reasonable request. The dream reports, however, will not be available as they have not been anonymized.

## Appendix A

“I am trapped with several people by Russians or the like, my boyfriend (“mein Freund”) is also there and they say that if we want us to be better, he should let me cut something off his body. I think of a finger or a toe, but my friend laughs, says it’s not a problem, hands me a red pocket knife, rolls up my left pant leg and says I should cut his leg below his knee. I imagine doing this, I’m desperate, try to talk him out of it. He just laughs, doesn’t see the seriousness of the situation. I don’t cut, something with a big black bird that can talk comes up.” Dream example (partner)

“I visited my ex-boyfriend. Actually, I just wanted just to say “hello” and therefore expected him to be very angry if I couldn’t spend that much time with him. But amazingly, he took it easy and more or less ignored my being stressed.” Dream example (ex-partner)

“I was in a large, bright tent with a large bed. A former classmate of mine lived there, but with whom I never had had anything and whom I actually never found particularly attractive, but had nothing directly against him. We had a really good chat, then he told me I was prettier than a former classmate of mine who I never liked. Then he touched my arm more several times during the conversation and then kiss me on the cheek. I liked him very much, but it occurred to me for a moment that I didn’t understand myself because I was never interested in him. I also thought about what the others who knew us would think when they found out we were together. But I quickly pushed the thoughts away and kissed him too. We slept together and I thought it was really nice.” Dream example (fictive partner)
